# Pediatric cartilaginous lateral femoral condyle Hoffa fracture: a case report and review of the literature

**DOI:** 10.1186/s12887-023-04448-6

**Published:** 2023-12-11

**Authors:** A. de Beer, M. J. Brown

**Affiliations:** 1 School of Medicine University of St. Andrews, N Haugh, St Andrews, KY16 9TF UK; 2 Connecticut Children’s Sports Medicine, 399 Farmington Ave, Farmington, CT 06032 UK

**Keywords:** Hoffa fracture, Arthroscopic fracture fixation, Bio-compression screws

## Abstract

**Introduction:**

Hoffa fractures are challenging coronally-oriented articular injuries of the femoral condyle. These fractures are rare in adults and extremely rare in the skeletally immature, with few cases reported in literature. To prevent mal- or non-union, Hoffa fractures require prompt surgical stabilisation with anatomic reduction and internal fixation.

**Case report:**

We discuss the case of a lateral distal femoral condyle cartilaginous Hoffa fracture in a ten-year-old male patient. The patient presented after a football non-contact “twist and pop” injury with radiographic imaging described as an osteochondritis dissecans lesion. An MRI was obtained which demonstrated a lateral distal femoral condyle osteochondral fracture. An operative plan was formulated to perform arthroscopic reduction and bio-compression screw fixation to minimize damage to the physis and surrounding tissues. Hyperflexion of the knee allowed for anatomic fracture reduction with the placement of 2 bio-compression screws serving as maintenance of fixation. The patient did well postoperatively and returned to full activity after 6 months.

**Conclusion:**

Hoffa fractures in the pediatric population are rare and can occur not only through bone but also through the thick chondral layer in younger patients. These are extremely difficult to diagnose through X-Ray alone. The prompt use of MRI imaging allows for operative fixation in a timely fashion, while an arthroscopic-only approach allows for minimal tissue damage. With an appropriate fracture type, hyper-flexion reduces and stabilizes the fracture, permitting the placement of minimally invasive bio-compression fixation.

## Introduction

Hoffa fractures are a rare coronal plane fracture of the distal femoral condyle. The fracture can involve one or both condyles, with lateral Hoffa fractures proving more common due to physiological genu valgum [1]. Hoffa fractures are more frequently described in adults, while coronal condyle fractures in pediatric patients are exceedingly rare. Distal femoral physeal fractures account for less than 1% of fractures in children and 1–6% of all physeal injuries [2, 3]. In adults and children the mechanism of injury is not sufficiently understood, but is commonly associated with high velocity trauma [1]. However, sports injuries and low energy trauma have been documented in literature [4, 5].

Non-operative treatment is associated with a high rate of complications as lack of soft tissue attachment threatens blood supply to the fracture fragment [6]. Malunion, non-union, avascular necrosis, premature arthritis, and knee stiffness have been reported with non-operative management. To mitigate against these risks, surgical stabilization with anatomic reduction and internal fixation is the treatment of choice. Arthroscopy-guided fixation is advantageous as soft tissue dissection is avoided and both blood loss and operative time are reduced, resulting in faster recovery and earlier mobilization [7].

This article discusses the case of a 10-year-old male patient presenting with a lateral condylar chondral Hoffa fracture. The presented case was successfully treated by arthroscopically assisted internal fixation. While a number of pediatric Hoffa fractures have reported in literature to date, none have been managed using an all-arthroscopic reduction and fixation technique discussed in this article. Consent was obtained from the patient’s parents to publish his case, including clinical images of the patient.

## Case report

**Fig. 1:**
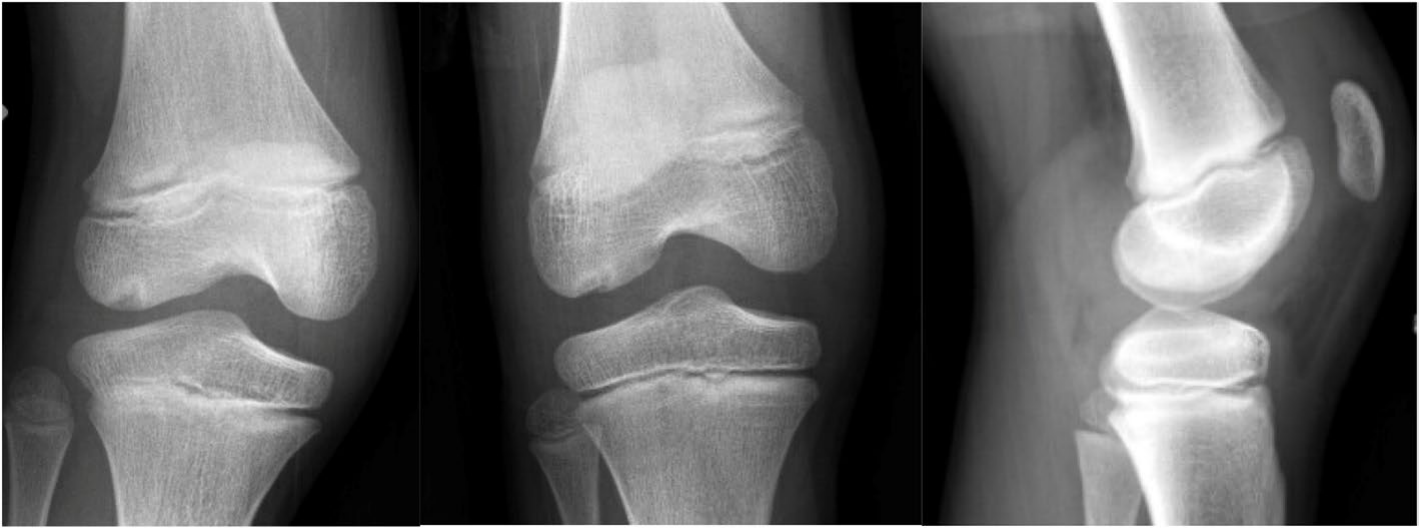
Initial XR demonstrating an apparent lateral femoral condyle Osteochondritis Dissesicans lesion

**Fig. 2:**
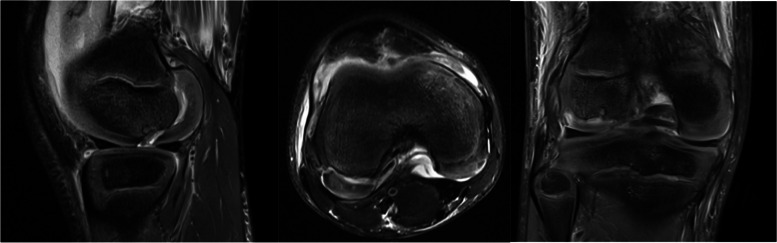
Saggital plane cartilaginous lateral femoral condyle Hoffa fracture revealed

The patient is a 10-year-old male who initially presented to the emergency room after suffering a right knee injury playing football. He described a noncontact, twisting and pivoting mechanism and felt a “pop” with pain. Afterwards the patient was unable to bear weight. He was neurovascularly intact with a significant effusion and decreased range of motion noted in the ER. Imaging demonstrated an osteochondral defect at the weightbearing lateral femoral condyle measuring 0.9 cm medial to lateral with a depth of 0.4 cm (Fig. 1). The patient was referred in an urgent basis to the sports medicine department, where he was seen within 3 days. An MRI of the right knee was ordered and was referred to an orthopaedic surgeon after the results were obtained (Fig. 2). Surgery to address the osteochondral fracture of the lateral condyle of the femur was performed within 3 weeks of the initial injury.

**Fig. 3:**
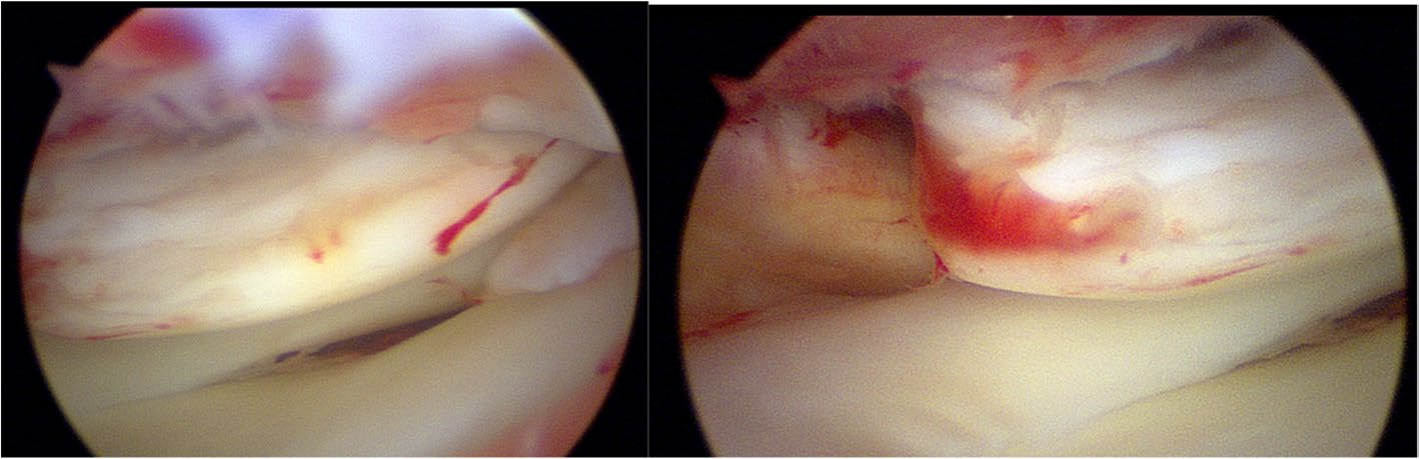
Mobile cartilaginous Hoffa fracture demonstrated

**Fig. 4:**
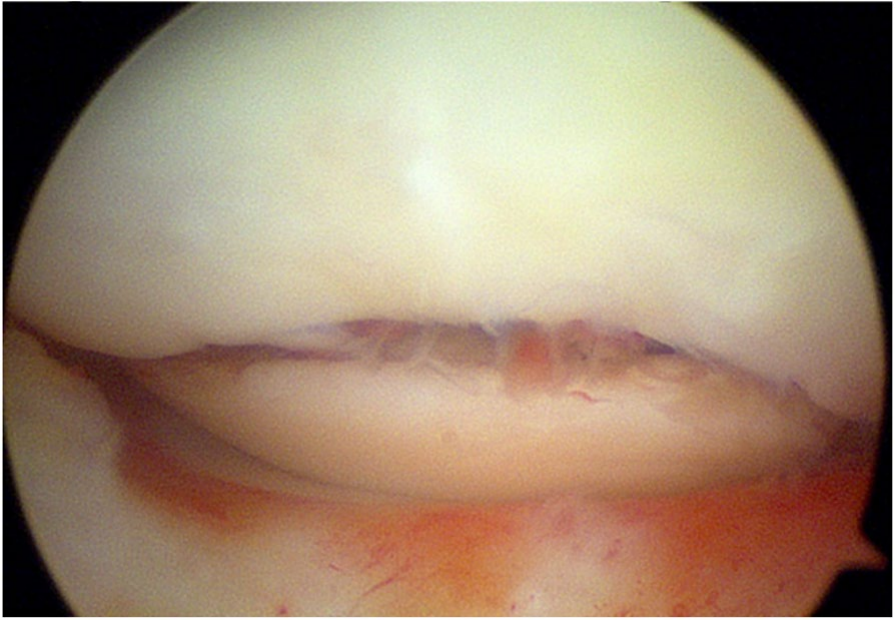
Fracture reduced with hyper-flexion (120 degrees)

**Fig. 5:**
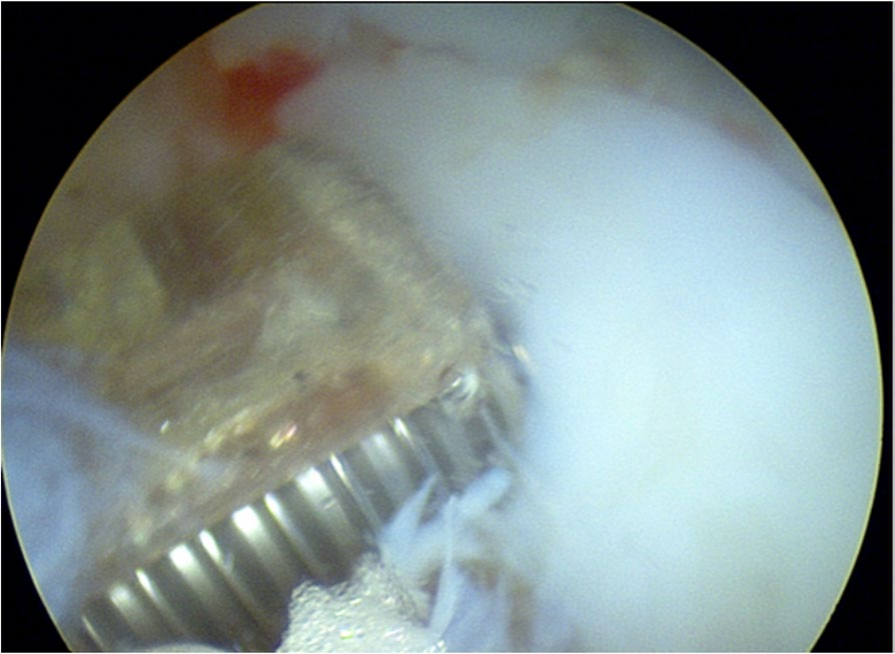
Initial reduction and fixation

**Fig. 6:**
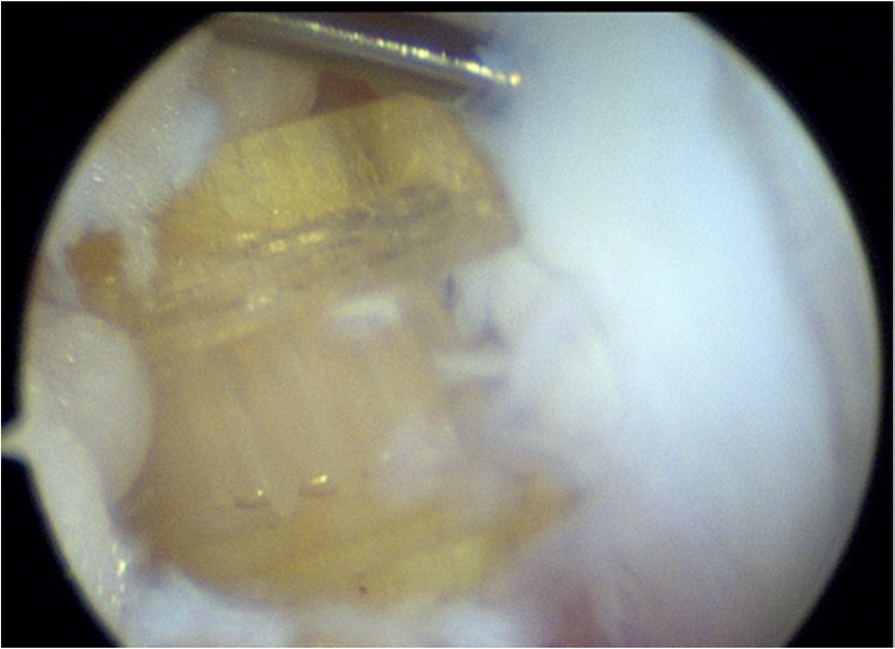
Fixation below K-wire

**Fig. 7:**
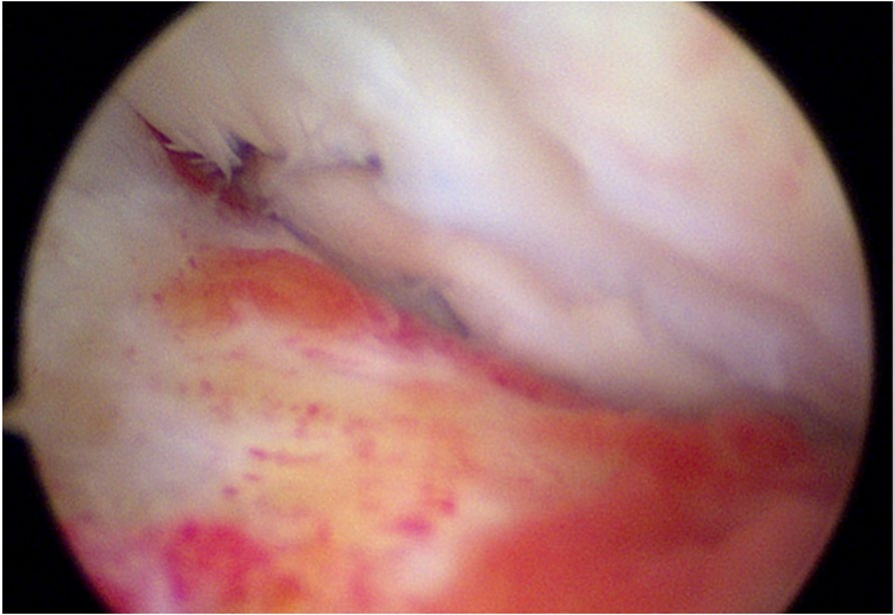
Anatomic fixation in flexion after 2 screw placement

The patient was positioned supine on the table with the right leg flexed and a bolster placed under the heel. A tourniquet was placed on the upper right thigh. Arthroscopy was performed through the usual anteromedial and anterolateral portals. Initially, the hemarthrosis was evacuated and a diagnostic arthroscopy was performed. The cruciates and menisci were found to be intact. The lateral femoral condyle chondral fragment was mobile, and we were able to completely free it from surrounding tissue and debrided the anterior and middle aspects free of clots and intervening soft tissue using a freer and shaver (Fig. 3). We then were able to flex the knee to 120 degrees, which reduced the fracture well and positioned the anterior aspect of the fracture fragment in line with the portal sites (Fig. 4). We made adjustments in the reduction with a freer and then used a 1.62 mm K wire placed only into the fracture fragment as a joystick to both reduce and then buried the K wire into the underlying bony condyle to secure the fragment. A second similarly sized K wire was placed in the midbody of the fragment to maintain reduction. We drilled, tapped, and then placed a 3 mm × 23 mm Arthrex bio compression screw through the lateral portal into the more anterior aspect of the fragment (Fig. 5). We then made a third, more distal parapatellar portal to allow for direct access and placement of a 3 mm × 26 mm Arthrex bio compression screw into the midbody of the fragment (Fig. 6). Both screws had excellent bite and they were recessed under the level of the chondral surface. The K wires were removed, and the knee put through a full range of motion, which demonstrated preserved reduction and fixation of the fragment (Fig. 7).

**Fig. 8:**
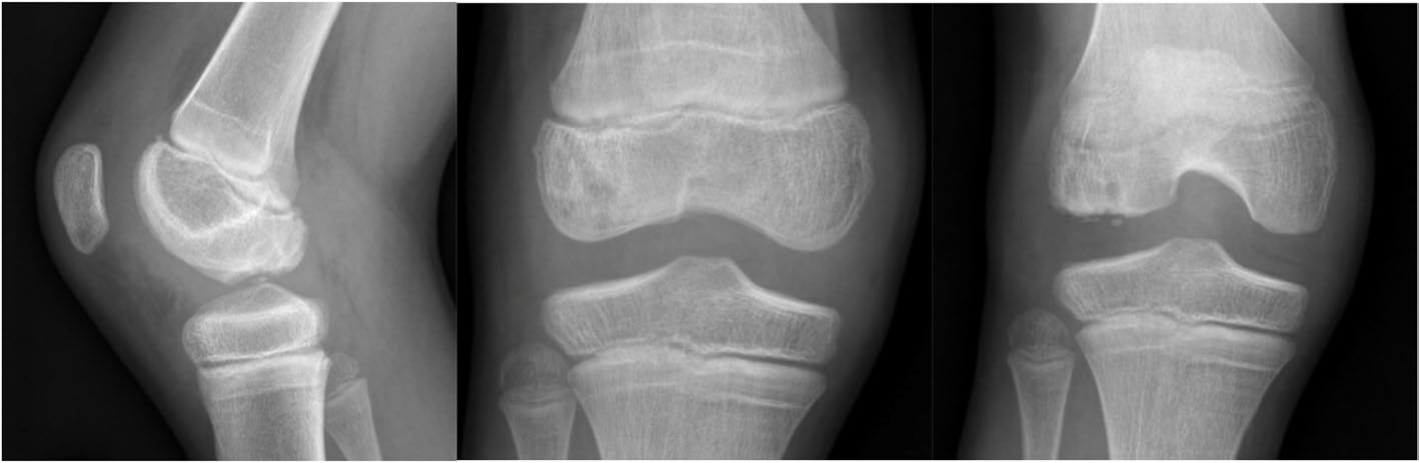
6 month XR images demonstrating interval osteochondral lesion healing

**Fig. 9:**
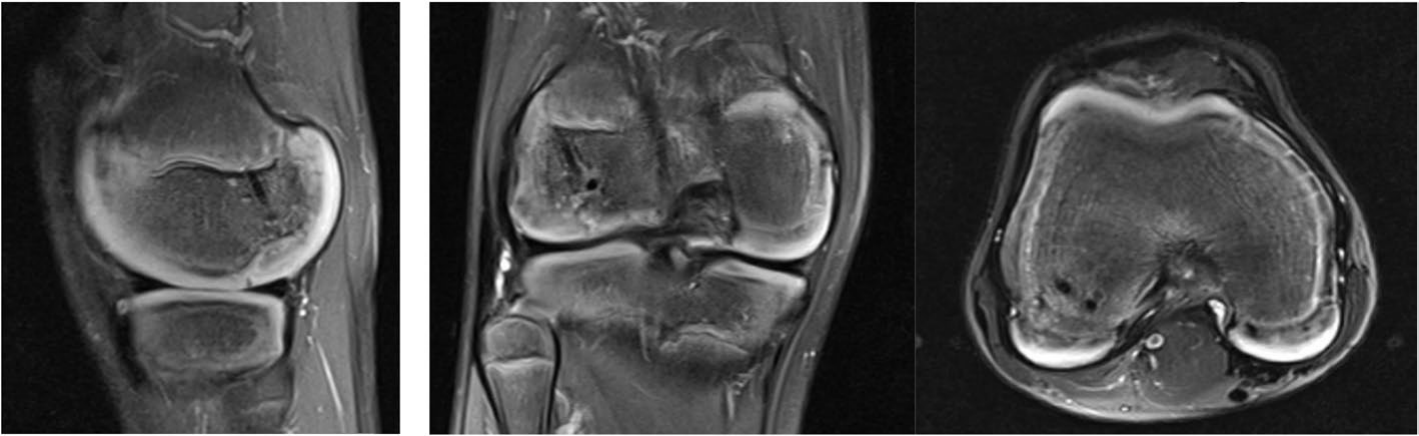
MRI 1 year after surgery demonstrating anatomic fixation and subsequent healing

The patient was allowed to be toe-touch weightbearing for balancing purposes for 4 weeks. During this period, he had knee range-of-motion (ROM) 0-30 degrees to allow for ground clearance using crutches. After 4 weeks he was advanced to partial weight bearing and his knee ROM increased to 0-90 degrees. The patient was involved with physical therapy starting 1 week after surgery with an initial focus on ROM within the previously delineated boundaries. At 6 weeks X-Rays (XR) were taken, which demonstrated early healing, full weight bearing resumed and the patient also began nonimpact strengthening physical therapy exercises. After 6 weeks the patient began riding a bicycle and swim-based therapy. The patient was also seen at 3- and 6-month timepoints, where he continued to increase his activity and his XR demonstrated continued healing and osseous ingrowth into the chondral fracture area. At 6 months the patient was cleared to return to all activities after images were obtained and he had cleared physical therapy (Fig. 8). An MRI was obtained at roughly 1 year to assess chondral healing (Fig. 9).

## Discussion

All previously published pediatric Hoffa fracture papers (15 case reports, Table 1) demonstrated that, among the pediatric age group, the most common coronal fracture is the lateral condyle, followed by bicondylar. Isolated medial condyle fractures are the most rare [19]. It has been hypothesized that the predominance of lateral condyle fractures is due to physiological genu valgum [7]. It has been suggested that the coronal fracture plane may be due to an axial load to the femoral condyle with the knee in 90 degrees or more flexion [20]. As a whole, however, with injury mechanisms as varied as low energy ‘twist-and-pop’ to high-energy motor vehicle accidents, the fracture cause is not well understood [10].

**Table 1:** Review of the Literature on Hoffa Fractures in the Pediatric Population

No.	Author and Year	Case	Fracture Location	Injury Mode	Surgical Fixation
1	McDonough et al., 2000 [8]	8-year-old child	Lateral femoral condyle (Non-union 5 years)	Road traffic accident	Open reduction and internal fixation and fixed with two posterior to anterior partially threaded cancellous lag screws without the usage of bone graft
2	Kumar et al., 2001 [9]	17-year-old female	Lateral femoral condyle	Fall from ladder	Open reduction and internal fixation with 2 anteroposterior lag screws
3	Flanagin et al., 2011 [4]	14-year-old male	Lateral femoral condyle	Wrestling	Arthroscopic evaluation followed by open arthrotomy and fixation with four headless screws
4	Tripathy et al., 2013 [10]	12-year-old child	Lateral femoral condyle	Fall while playing	Posterolateral approach of knee using two partially threaded AO cancellous lag screws
5	Potini et al., 2015 [11]	14-year-old male	Lateral femoral condyle	Direct blow over knee	Open reduction and rigid fixation with countersunk interfragmentary screws along with augmentation of allograft for the articular damage
6	Elazab et al., 2019 [12]	12-year-old male	Lateral femoral condyle	Motor vehicle accident	Diagnostic arthroscopy followed by open arthrotomy using anterolateral approach for excision of pseudoarthrosis with application of two reduction forceps, open reduction, and internal fixation of the fracture with lag screws
7	Lal et al., 2011 [7]	9-year-old child	Conjoint	Fall from height onto a flexed knee	Arthroscopy assisted fracture fixation with 4.5 mm anteroposterior cannulated cancellous screws
8	Kondreddi et al., 2014 [13]	17-year-old	Conjoint	Road traffic accident	Lateral parapatellar arthrotomy with 4 mm cancellous screws (two for each condyle anteroposteriorly through the non-articular surface)
9	Harna et al., 2017 [14]	7-year-old male	Conjoint	Hit by a speeding motor vehicle	Swashbuckler approach with 2.9 mm Herbert screw
10	Julfiqar et al., 2019 [15]	12-year-old male	Conjoint	Fall from height when his left knee in flexed position	Open reduction and intraepiphyseal internal fixation using 4.5 mm cannulated cancellous screw and bone to tendon repair
11	Bali et al., 2011 [5]	12-year-old male	Medial femoral condyle	Motorbike accident while riding on a pillion	Subvastus approach with two large fragment, anterior to posteromedial cannulated screws
12	Salunke et al., 2015 [16]	16-year-old female	Medial femoral condyle	Car accident	Subvastus approach with 2 cannulated cancellous screws
13	AlKhalife et al., 2018 [17]	12-year-old male	Medial femoral condyle	Fall of heavy object over knee	Medial parapatellar approach
14	Ranjan et al., 2020 [18]	6-year-old female	Medial femoral condyle	Fall from 12 ft height	Medial parapatellar approach with 2 mediolateral, cannulated cancellous screws
15	Current Study, 2022	10-year-old male	Lateral Femoral condyle	Football non-contact injury	Arthroscopic fixation with biocompression screws

Diagnosis of a Hoffa fracture is challenging. Rigorous clinical examination with appropriate imaging is imperative for diagnosis in the skeletally immature. The lesion is often obscured on radiographs, causing such injuries to be missed, especially minimally displaced fractures. On anteroposterior radiographs, the intact anterior part of the condyle can obscure the fracture. On lateral radiographs, the femoral condyles overlap, and potentially obscure the fracture [20]. The oblique view provides better visualization of minimally displaced fractures [13]. While CT scanning is the gold standard for characterization and diagnosis of Hoffa fractures [18], due to the radiological exposure risk in younger patients, MRI is preferred. In this case, the initial radiographic report described an osteochondritis dissecans lesion, which led to the Hoffa fracture being later diagnosed on an MRI scan. Our case exemplifies challenges in diagnosis of Hoffa fractures and the value of appropriate MRI imaging.

In 1978, Letenneur et al. [21] classified Hoffa fractures into 3 types. Each correspond to the distance of the fracture line from the posterior femoral cortex. Classification is clinically significant as it delineates the relationships between the ligaments, soft tissue and fracture line. Type I is a fracture parallel to the posterior femoral cortex involving the entire posterior condyle. Type II is similar, but with a more posterior fracture line. Type II is further divided into thirds, by distance - classified as A, B and C. Type III has an oblique fracture line and is either lateral, medial or conjoined bicondylar. Hoffa fractures are classified under the AO classification as 33-B3, 33B3.2 when the fracture is unicondylar and 33B3.3 for bicondylar involvement [22]. In our case, as the fracture was only cartilaginous, neither Letenneur nor AO classification can be applied. However, the general classification is important as the more posterior the fracture plane lies, the greater difficulty in providing arthroscopic-only treatment.

Once the appropriate diagnosis has been reached, it is generally accepted that surgical stabilization is needed to achieve satisfactory long-term function. This is due to the difficulty of successful closed reduction with casting/splinting. In the cited literature there is minimal to no closed treatment due to the high risk for malunion or non-union with chronic pain and disability [6]. The other cases, all treated in an open fashion, necessitated a metal screw-based fixation with conscious avoidance of the physis to minimize both growth risks and the need for future screw removal. In our case, the minimally invasive approach and use of bio-compression screws allowed for direct reduction and perpendicular fracture fixation without the worry of physeal violation and possible growth anomalies, let alone future surgical hardware removal.

Due to decreased post-operative morbidity, we advocate for arthroscopic-guided fixation over open fixation where appropriate and within the surgeon’s skill-set. Reasons include arthroscopic-guided fixation avoiding an extended incision with resulting soft tissue disruption, reducing scaring and stiffness [23]. Excellent intra-articular visualization from arthroscopy aids both diagnosis and treatment of the Hoffa fracture alongside other intra-articular injuries. However, the arthroscopic technique is not possible in all patients. Goel A et al. note that consideration should be given to pre-consenting patients for the backup plan of an open reduction and internal fixation [22]. Arthroscopic technique requires a high degree of skill and experience; despite this, the merits outweigh the limitations in the appropriate context.

## Conclusion

Hoffa fractures in the pediatric population are rare and can occur not only through bone but also through the thick chondral layer in younger patients. These are extremely difficult to diagnose through XR alone. Prompt use of MRI imaging allows for operative fixation in a timely fashion. An arthroscopic-only approach allows for minimal tissue damage. With an appropriate fracture type, hyper-flexion reduces and stabilizes the fracture, allowing for the placement of minimally invasive biocompression fixation.

## Data Availability

The datasets used and/or analyzed during the current study available from the corresponding author on reasonable request.

## Abbreviations

XR: X-Ray
ROM: Range-of-motion
MRI: Magnetic resonance imaging
